# Biomarker modeling of Alzheimer’s disease using PET-based Braak staging

**DOI:** 10.1038/s43587-022-00204-0

**Published:** 2022-04-25

**Authors:** Joseph Therriault, Tharick A. Pascoal, Firoza Z. Lussier, Cécile Tissot, Mira Chamoun, Gleb Bezgin, Stijn Servaes, Andrea L. Benedet, Nicholas J. Ashton, Thomas K. Karikari, Juan Lantero-Rodriguez, Peter Kunach, Yi-Ting Wang, Jaime Fernandez-Arias, Gassan Massarweh, Paolo Vitali, Jean-Paul Soucy, Paramita Saha-Chaudhuri, Kaj Blennow, Henrik Zetterberg, Serge Gauthier, Pedro Rosa-Neto

**Affiliations:** 1 Translational Neuroimaging Laboratory, McGill Research Centre for Studies in Aging, Douglas Mental Health Institute, Le Centre intégré universitaire de santé et de services sociaux (CIUSSS) de l’Ouest de l’Île de Montréal, Montreal, Quebec Canada; 2 Department of Neurology and Neurosurgery, Faculty of Medicine, McGill University, Montreal, Quebec Canada; 3 Department of Psychiatry, University of Pittsburgh School of Medicine, Pittsburgh, PA USA; 4 Clinical Neurochemistry Laboratory, Sahlgrenska University Hospital, Mölndal, Sweden; 5 Department of Psychiatry and Neurochemistry, Institute of Neuroscience and Physiology, The Sahlgrenska Academy, University of Gothenburg, Gothenburg, Sweden; 6 King’s College London, Institute of Psychiatry, Psychology and Neuroscience, Maurice Wohl Institute Clinical Neuroscience Institute, London, UK; 7 Biomedical Research Unit for Dementia at South London, NIHR Biomedical Research Centre for Mental Health and Maudsley NHS Foundation, London, UK; 8 Department of Math & Statistics, University of Vermont, Burlington, VT USA; 9 Department of Neurodegenerative Disease, UCL Institute of Neurology, Queen Square, London, UK; 10 UK Dementia Research Institute at UCL, London, UK

**Keywords:** Alzheimer's disease, Diagnostic markers, Ageing

## Abstract

Gold-standard diagnosis of Alzheimer’s disease (AD) relies on histopathological staging systems. Using the topographical information from [^18^F]MK6240 tau positron-emission tomography (PET), we applied the Braak tau staging system to 324 living individuals. We used PET-based Braak stage to model the trajectories of amyloid-β, phosphorylated tau (pTau) in cerebrospinal fluid (pTau_181_, pTau_217_, pTau_231_ and pTau_235_) and plasma (pTau_181_ and pTau_231_), neurodegeneration and cognitive symptoms. We identified nonlinear AD biomarker trajectories corresponding to the spatial extent of tau-PET, with modest biomarker changes detectable by Braak stage II and significant changes occurring at stages III–IV, followed by plateaus. Early Braak stages were associated with isolated memory impairment, whereas Braak stages V–VI were incompatible with normal cognition. In 159 individuals with follow-up tau-PET, progression beyond stage III took place uniquely in the presence of amyloid-β positivity. Our findings support PET-based Braak staging as a framework to model the natural history of AD and monitor AD severity in living humans.

## Abstract (web summary)

The authors used PET imaging to stage individuals according to the Braak neuropathological system for Alzheimer’s disease. PET stage was associated with biomarker and cognitive changes, highlighting the potential to stage Alzheimer’s disease in living people.

## Main

In several areas of medicine, measurement of disease severity is necessary to evaluate clinical diagnostic accuracy, guide patient management, evaluate treatment response and design therapeutic trials. Staging systems provide a framework for measuring the severity of a disease based on the reliable identification of important points in the disease course^1^. Disease stages are associated with clinical symptoms as well as prognosis and hence are important in guiding patient management. Staging systems also provide a framework for monitoring biomarker changes in relation to the natural history of a disease.

AD is characterized by the accumulation of amyloid-β and tau pathologies, followed by neurodegeneration and cognitive decline. Widely accepted pathobiological models of AD suggest that amyloid-β and tau pathologies accumulate over two decades in the absence of symptoms^2–4^. The recently proposed biological research framework for AD permits the identification of AD in living humans based on abnormal concentrations of amyloid-β and tau biomarkers^5^. In contrast, the postmortem identification of AD is based on staging systems that take into account the anatomical localization of neuropathology^6,7^. One such staging system, proposed by Braak and Braak, is based on the anatomical localization of tau neurofibrillary tangles^8–10^. This hierarchical system stages AD according to tau aggregation in the transentorhinal cortex (stage I), entorhinal cortex and hippocampus (stage II), inferior temporal neocortex (stage III), association cortices (stages IV and V) and primary sensory cortices (stage VI). Although Braak staging is part of the gold-standard diagnostic workup for AD at autopsy^6,7^, it remains a largely histopathological construct and has yet to be integrated into the definition of AD in vivo^5,11–14^.

Here using a tau-PET ligand with subnanomolar affinity for tau neurofibrillary tangles, we leverage the topographical information conferred from PET imaging to apply the Braak neurofibrillary tangle staging system to living humans. We subsequently used Braak stage to model the progression of AD biomarker abnormalities in relation to the spatial topography of tau-PET. We report relationships between PET-based Braak stage and dynamic biomarker changes across the course of AD, including stage-specific associations with multiple pTau phosphorylation sites in cerebrospinal fluid (CSF) and plasma. Braak stages were furthermore associated with the evolution and plateau of amyloid-β measured with PET and CSF. Early Braak stages were associated with isolated memory impairment and sparing of other cognitive domains, whereas later Braak stages were associated with the severity of dementia. Longitudinal analyses suggested that PET-based Braak stages progress in a sequential fashion, with progression beyond stage III requiring the presence of abnormal amyloid-β. Our results support PET-based Braak staging as a framework to model the natural history of AD, as well as monitor AD severity from presymptomatic to clinical dementia phases.

## Results

We applied the histopathological Braak neurofibrillary tangle staging model to 179 cognitively unimpaired (CU) older adults, 80 individuals with mild cognitive impairment (MCI), and 65 individuals with Alzheimer’s clinical syndrome. Demographic, clinical and summary biomarker characteristics of the samples are reported in Table 1. Mean age of participants in the study was 70.05 (s.d. = 7.95) years; 60% were female. Based on the anatomical localization of tau-PET abnormality within brain regions described by Braak and Braak^8–10^, individuals were assigned a PET-based Braak stage ranging from 0 (no detectable tau-PET abnormality) to VI (tau-PET abnormality extending to primary sensory areas).

**Table 1:** Demographic, clinical and summary biomarker characteristics of the samples

	CU	MCI	*P* value	Alzheimer syndrome	*P* value
Number of individuals	179	80	**–**	65	**–**
Age (y), mean (s.d.)	71.12 (7.18)	70.34 (8.1)	0.45	66.71 (9.8)	0.0001
Female, *n* (%)	113 (63)	43 (53)	0.15	41 (63)	0.99
Education (y), mean (s.d.)	15.50 (3.82)	15.06 (3.61)	0.38	14.28 (4.03)	0.03
APOE *ε4* carriers, *n* (%)	50 (28)	32 (40)	0.25	32 (49)	0.0007
MMSE, mean (s.d.)	29.12 (1.48)	27.98 (1.75)	<0.0001	19.78 (6.23)	<0.0001
Neocortical [^18^F]AZD4694 SUVR, mean (s.d.)	1.45 (0.38)	2.01 (0.63)	<0.0001	2.40 (0.68)	<0.0001
Temporal Meta-ROI [^18^F]MK6240 SUVR, mean (s.d.)	1.06 (0.15)	1.46 (0.55)	<0.0001	2.82 (1.03)	<0.0001
Hippocampal volume (cm^3^), mean (s.d.)	3.54 (0.41)	3.29 (0.44)	<0.0001	2.84 (0.51)	<0.0001

*P* values reported are for comparisons to cognitively unimpaired subjects. *P* values indicate values assessed with two-sided independent-samples *t* tests for each variable except sex and APOE *ε4* status, where contingency *χ*^2^ tests were performed. No adjustments were made for multiple comparisons for participant demographics. APOE *ε4*, apolipoprotein epsilon 4; MMSE, Mini-Mental State Examination; ROI, region of interest; SUVR, standardized uptake value ratio.

### Tau aggregation follows hierarchical pattern

**Fig. 1:**
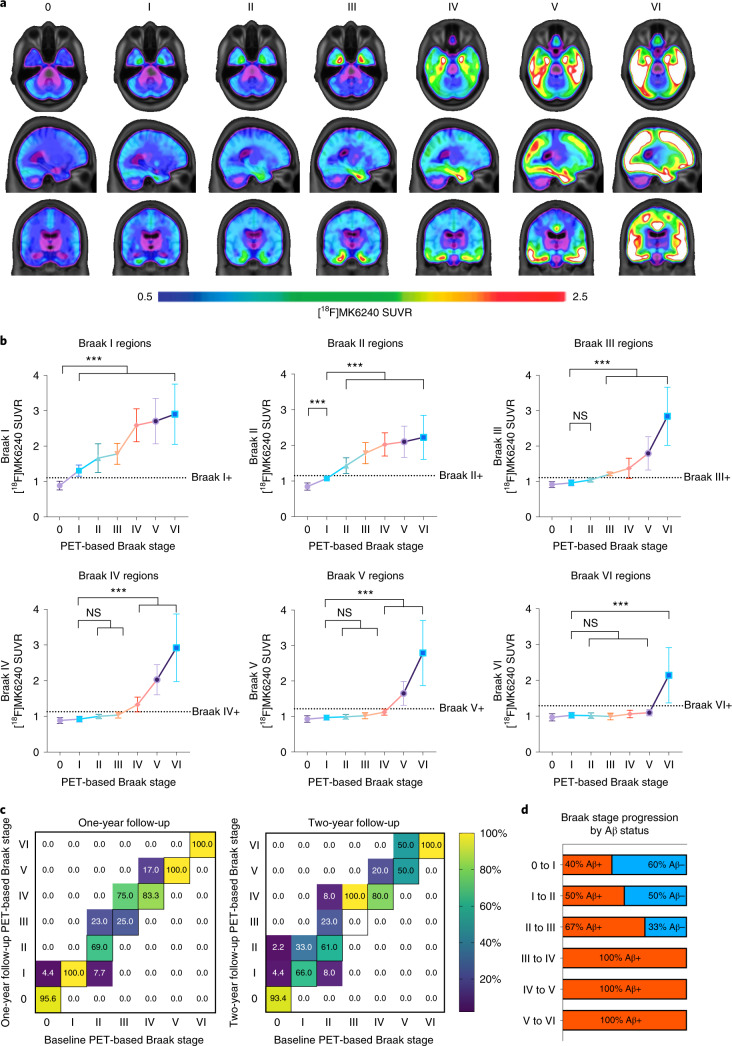
PET-based Braak staging captures variability in magnitude and topography of pathological tau. **a**, Average [^18^F]MK6240 SUVRs across the whole brain for all participants grouped according to PET-based Braak stage. Individuals at stage 0 did not have detectable tau abnormality in regions comprising any Braak stage. At stage I, tau abnormality is confined to the transentorhinal cortex. Braak stages V and VI are characterized by high magnitude of tau pathology, with stage VI extending to primary sensory cortices. **b**, Regional accumulation of tau-PET conforms to hierarchical histopathological tau staging model. Higher levels of tau-PET SUVR in the transentorhinal cortices are observed at all subsequent stages (family-wise error (FWE)-corrected *P* < 0.001 for all). In the entorhinal and hippocampal cortices (Braak II regions), tau-PET is abnormal only in individuals assigned to PET-based Braak stage II and above and increased with advancing PET-based Braak stage. Overall, cortical regions remain largely spared until the corresponding Braak stage is reached. Only individuals assigned to stage VI had statistically different tau-PET SUVR in regions comprising Braak VI. Dashed lines represent stage-specific cutoffs for tau-PET in each Braak stage. Group means are represented by shapes, and error bars represent standard deviation. Stage I is represented by the purple circle, stage II is represented by the blue square, stage III is represented by the green triangle, stage IV is represented by the orange inverted triangle, stage V is represented by the hollow purple circle and stage VI is represented by the hollow blue square. Successive Braak stages are connected by a solid line, which carries the color of the previous stage. ***P < 0.001; two-sided analysis of variance (ANOVA) corrected for multiple comparisons using Dunnett’s correction (*n* = 324). **c**, Longitudinal stability and progression of Braak stage. Percentages along the diagonal indicate proportion of individuals at each Braak stage who did not change Braak stage over the follow-up period. Deviations from the diagonal indicate a change in Braak stage; percentages above the diagonal midline indicate Braak stage progression, and percentages below the diagonal midline likely represent misclassifications. **d**, Stage-specific associations between amyloid-β positivity and PET-based Braak stage progression. Aβ, amyloid-β; NS, not significant.

**Extended Data Fig. 1:**
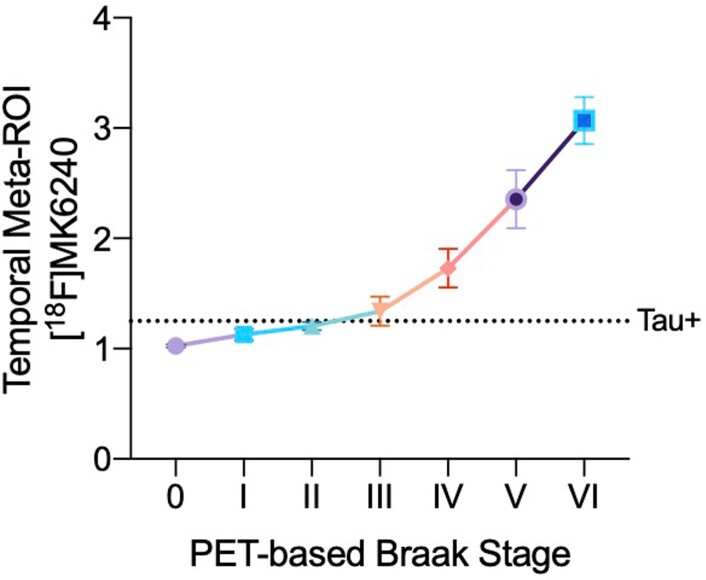
Tau-PET summary measures increase with respect to PET-based Braak stage. Tau-PET in the temporal meta-ROI increases nonlinearly with advancing Braak stage (p < 0.0001; ANOVA two-sided ANOVA corrected for multiple comparisons using Dunnett’s correction; n = 324 individuals). The dashed line indicates a previously validated threshold for tau positivity using the temporal meta-ROI. This tau positivity threshold is located between the group-level means of Braak stages II and III, supporting the notion that tau positivity is associated with tau spread outside the medial temporal lobe. Group means are represented by shapes, error bars represent standard deviation.

**Extended Data Fig. 2:**
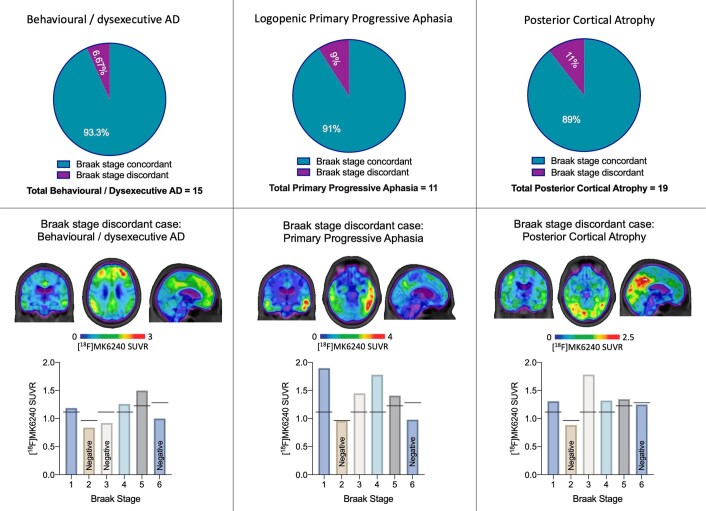
Atypical clinical phenotypes of AD are largely compatible with Braak staging model. **Top**: proportion of cases with established focal cortical presentations of AD that fit (turquoise) and do not fit (purple) with the Braak staging system. **Middle**: Tau-PET images of representative Braak-discordant cases are displayed in the middle. **Bottom**: [^18^F]MK6240 Tau-PET uptake in each Braak stage of discordant case presented. Absence of tau abnormality in Braak stage II (entorhinal cortex and hippocampus) was the most common reason for Braak discordance in atypical focal cortical presentations of AD, and may be associated with MTL-sparing AD. Relative tau-PET uptake within certain Braak stages may be associated with relative predominance of specific cognitive impairment and clinical presentation. Individuals with nonamnestic dementia phenotypes were included in the main analyses. The individual focal cortical syndrome cases displayed above were classified as Braak stage V.

Figure 1a displays the average whole-brain [^18^F]MK6240 tau-PET SUVRs grouped by PET-based Braak stage. Stage I is characterized by detectable tau pathology in the transentorhinal cortex and the absence of abnormal tau-PET in any other region. Individuals at PET-based Braak stage II had tau abnormality in the entorhinal cortex and hippocampus, in addition to involvement of stage I. Stages III and IV displayed increased involvement of earlier stages as well as larger occupation of the temporal neocortex. Individuals classified as PET-based Braak stage V had more extended involvement of association cortices, whereas individuals classified as PET-based Braak stage VI had tau abnormality extending into primary sensory areas. Figure 1b displays the hierarchical aggregation characteristic of Braak stages, in which tau abnormality at later Braak stages is only observed in the presence of tau abnormality in earlier Braak stages. All individuals classified as a nonzero Braak stage had tau abnormality in stage I regions. Similarly, all individuals stage II and above had tau abnormality in stage II regions. Individuals at early stages (i.e., I–II) did not demonstrate tau abnormality at later stages; however, tau abnormality in later stages occurred in the presence of abnormality at earlier stages, suggesting that tau accumulation follows a hierarchical pattern. Although PET-based Braak stages were defined based on the anatomical localization of tau-PET, the magnitude of tau-PET SUVR in earlier stages also increased with each successive stage (*P* < 0.001 for all stages; summary statistics presented in Supplementary Table 1). Braak stage was also associated with tau-PET in the temporal meta-ROI (*P* < 0.0001; Extended Data Fig. 1), a common summary tau-PET outcome measure. We next investigated tau-PET patterns in individuals with atypical clinical phenotypes of AD (behavioral predominant, language predominant and visuospatial predominant). We observed that the Braak staging model was largely compatible with a variety of clinical presentations of AD (Extended Data Fig. 2). In atypical AD phenotypes, absence of tau abnormality in Braak stage II was the most common feature of tau-PET patterns that did not conform to the hierarchical Braak staging model.

In longitudinal analyses of 159 individuals with baseline and follow-up tau-PET data, PET-based Braak stage at follow-up either remained stable or progressed sequentially. Most individuals remained at the same Braak stage over 1 year (92%; Fig. 1c) and over 2 years (88%). One individual (0.7%) changed from Braak stage II to I, potentially indicating a misclassification. In instances where a change in PET-based Braak stage was observed, an increase of one Braak stage was the most common outcome (7.9% at 1 year, 10% at 2 years). Over 2 years, one individual progressed from Braak stage 0 to II, and one individual progressed from Braak stage II to IV. These individuals who progressed two Braak stages over 2 years (2%) were nonetheless positive at the intermediary Braak stages. Figure 1d represents the proportion of individuals who progressed to a higher PET-based Braak stage according to baseline amyloid-PET status. Amyloid-β negativity was compatible with progression from PET-based Braak stage 0 to I and from I to II. Amyloid-β negativity was also compatible with progression from Braak stage II to III, although it should be noted that some amyloid-β-negative individuals who progressed from Braak stage II to III were very close to the threshold for amyloid-β positivity. Progression from PET-based Braak stage III and beyond took place uniquely in the presence of amyloid-β positivity.

### Braak stages evolve according to global amyloid-PET uptake

**Fig. 2:**
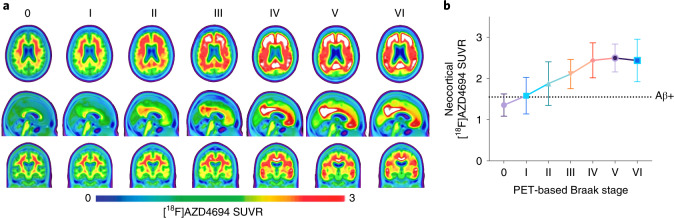
Amyloid-PET evolution with respect to Braak stage. **a**, Average [^18^F]AZD4694 amyloid-PET SUVRs across the whole brain grouped according to PET-based tau Braak stage. Each set of images represents the average amyloid-PET of individuals grouped according to their tau-PET scan. **b**, Neocortical amyloid-PET uptake increases and then plateaus with advancing Braak stage. The dashed line indicates a previously validated threshold for amyloid-PET positivity. Individuals at PET-based Braak stage 0 were often amyloid-β negative. Neocortical amyloid-PET SUVRs were higher at PET-based Braak stages I and II, although both stages were compatible with amyloid-β positivity and amyloid-β negativity. Individuals at Braak stage III and higher were almost exclusively amyloid-β positive. From Braak stage IV onwards, a plateau of amyloid-PET uptake was observed. Group means are represented by shapes, and error bars represent s.d. Summary statistics for all amyloid-PET comparisons are reported in Supplementary Table 2; two-sided ANOVA with Dunnett’s correction for multiple comparisons (*n* = 324).

**Extended Data Fig. 3:**
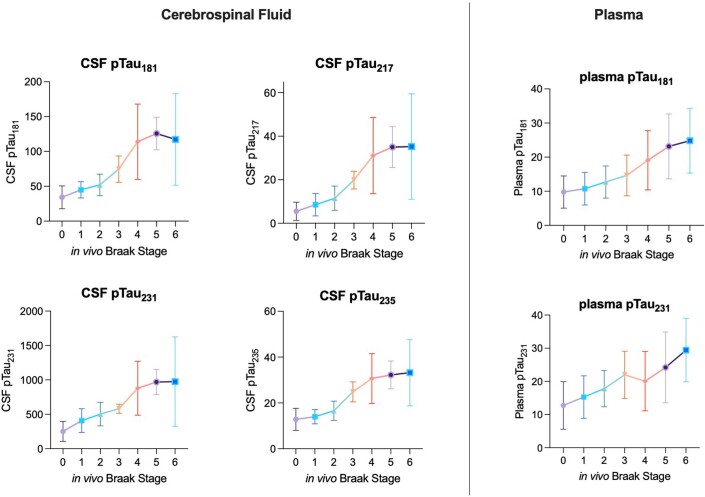
Phosphorylated tau species curves displayed with 95% Confidence Intervals. **Left**: CSF biomarkers of pTau epitopes increase with respect to PET-based Braak stages. For all CSF pTau epitopes, modest differences were observed at early Braak stages, likely reflecting the fact that only subtle pathological changes are occurring in the cerebral cortex. No differences were observed between stages V and VI for any CSF pTau measures (n = 189 individuals with lumbar punctures). **Right**: Plasma biomarkers of pTau181 and pTau231 increase with advancing PET-based Braak stage. Despite stepwise increases at the group level, large within-stage variability exists for each fluid pTau biomarker in each Braak stage. Means are represented by shapes and error bars indicate 95% CIs (n = 292 individuals with plasma pTau assessments).

Figure 2 displays the average whole-brain amyloid-PET SUVRs of individuals categorized according to their PET-based Braak stage. Most individuals at PET-based Braak stage 0 were amyloid-β negative. Early Braak stages (0–II) were compatible with both amyloid-β positivity and amyloid-β negativity, whereas late Braak stages were observed almost exclusively in amyloid-β-positive individuals. Continuous measurements of amyloid-PET SUVR demonstrated a rising of amyloid-PET with increases in early Braak stages and a plateau of amyloid-PET SUVR starting at Braak stage IV. When dichotomizing amyloid-PET SUVR into positive and negative categories, *χ*^2^ analyses revealed that the proportion of amyloid-β positivity versus negativity differed between early Braak stages (*P* < 0.01 for 0 versus I, 0 versus II and II versus III) but did not differ between stages IV–VI. Summary statistics for all amyloid-PET comparisons are provided in Supplementary Table 2. Similar results were observed when evaluating CSF concentrations of amyloid-β using the Aβ42/Αβ40 ratio (Extended Data Fig. 3 and Supplementary Table 3).

### PET-based Braak stages indicate the magnitude of pathological tau hyperphosphorylation

**Fig. 3:**
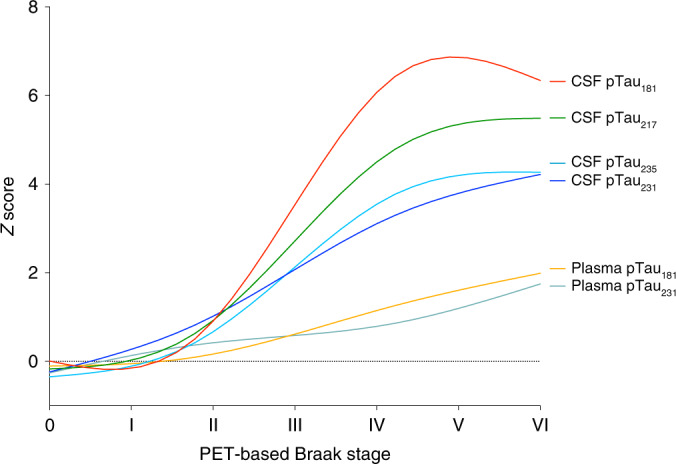
PET-based Braak stages reflect the evolution of soluble pTau species. Measures of four soluble pTau epitopes demonstrate differential evolution with respect to PET-based Braak stage. Magnitude of CSF pTau changes mirror the spatial extent of neurofibrillary tangle pathology. At Braak stage I, when tau abnormality is confined to the transentorhinal cortex, there are no statistically significant differences in any CSF or plasma pTau measures with individuals who are at Braak stage 0. At Braak stage II, small differences in the concentrations of CSF pTau_231_, pTau_217_, pTau_235_ and pTau_181_ are present. Starting at Braak stage III, when abnormal tau begins to accumulate outside the medial temporal lobe, CSF measures of pTau epitopes begin to show substantial increases. Larger differences in the magnitude of CSF pTau_231_ were observed at earlier stages, whereas larger differences in CSF pTau_181_ were observed at later stages. In contrast of CSF measurements, plasma measures of pTau show more modest increases across Braak stages, with plasma pTau_231_ becoming abnormal before plasma pTau_181_. The magnitude of pTau_181_ abnormality was largest at PET-based Braak stages IV–VI. CSF pTau measures exhibited plateaus at late Braak stages, whereas plasma pTau measures continued to increase. Curves were fit with locally estimated scatterplot smoothing (LOESS) regression. Summary statistics for all pTau comparisons are reported in Supplementary Table 3. Biomarker curves with 95% confidence intervals are displayed in Extended Data Fig. 3.

PET-based Braak stages reflected the evolution of four distinct soluble pTau epitopes in CSF (Fig. 3). Small yet detectable differences in CSF measures of pTau_181_, pTau_217_, pTau_231_ and pTau_235_ could be observed at PET-based Braak stage II (tau abnormality in entorhinal cortex and hippocampus; FWE-corrected *P* < 0.05 for all phosphorylation sites), but not stage I, when tau abnormality is confined to the transentorhinal cortex. Larger CSF pTau differences for all phosphorylation sites could be observed starting at stage III, when tau pathology begins to accumulate outside of the medial temporal lobe (FWE-corrected *P* < 0.05 for all phosphorylation sites). Starting at stage IV, CSF concentrations of pTau_181_, pTau_217_, pTau_231_ and pTau_235_ began to plateau. In addition to overall trajectories, we observed phosphorylation site-specific differences in the magnitude of CSF pTau abnormality with respect to PET-based Braak stage. CSF pTau_231_, closely followed by pTau_217_, demonstrated larger differences in magnitude at earlier Braak stages, whereas CSF pTau_181_ exhibited the largest differences at later Braak stages. Plasma concentrations of pTau_231_ were significantly different from stage 0 by PET-based Braak stage II. Plasma concentrations of pTau_181_ demonstrated significant differences from stage 0 starting at PET-based Braak stage IV. Similar to CSF concentrations, plasma pTau_231_ exhibited differences at earlier Braak stages, whereas plasma pTau_181_ exhibited larger differences at later Braak stages. In contrast to CSF concentrations, plasma pTau231 did not exhibit a plateau at later stages. Summary statistics for all pTau comparisons are provided in Supplementary Table 5. Fluid pTau biomarker curves represented with 95% confidence intervals are presented in Extended Data Fig. 3.

### Braak staging tracks AD symptoms from memory impairment to dementia

**Fig. 4:**
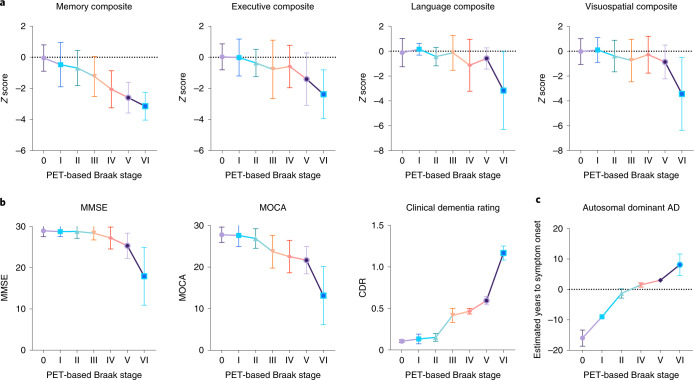
Early Braak stages are associated with isolated memory dysfunction and late Braak stages are associated with dementia severity. **a**, Memory, executive, language and visuospatial composite *Z* scores according to PET-based Braak stage (*n* = 291 individuals with neuropsychological evaluation available). Memory function begins to decline at Braak stage II, while other cognitive domains remain unaffected. Memory continued to decline with increasing Braak stage. Language and visuospatial domains remain relatively spared until late Braak stages. **b**, Summary cognitive assessments according to PET-based Braak stage. Little change in MMSE scores were observed from Braak stages 0 to III. Steeper declines in MMSE score were observed at Braak stages V and VI. Changes in MOCA score were detectable by stage IV. Braak stages 0–II were largely compatible with the absence of dementia as assessed by the CDR. Braak stages III and IV consisted almost exclusively of individuals with CDR = 0.5 (very mild dementia). Braak stage V and VI were incompatible with normal cognition, with Braak stage VI being associated with increased dementia severity (*n* = 324 with summary cognitive assessments). **c**, In a subsample of individuals with autosomal dominant AD (*PSEN1* mutation carriers; *n* = 14, total of 21 tau-PET scans including follow-ups), PET-based Braak stage was associated with estimated years to the onset of symptoms. Shapes indicate means, and error bars indicate s.d. Summary statistics for all cognitive outcome comparisons are reported in Supplementary Table 6. Statistics were conducted with two-sided ANOVA using Dunnett’s correction for multiple comparisons. *PSEN1*, Presenilin-1.

**Extended Data Fig. 5:**
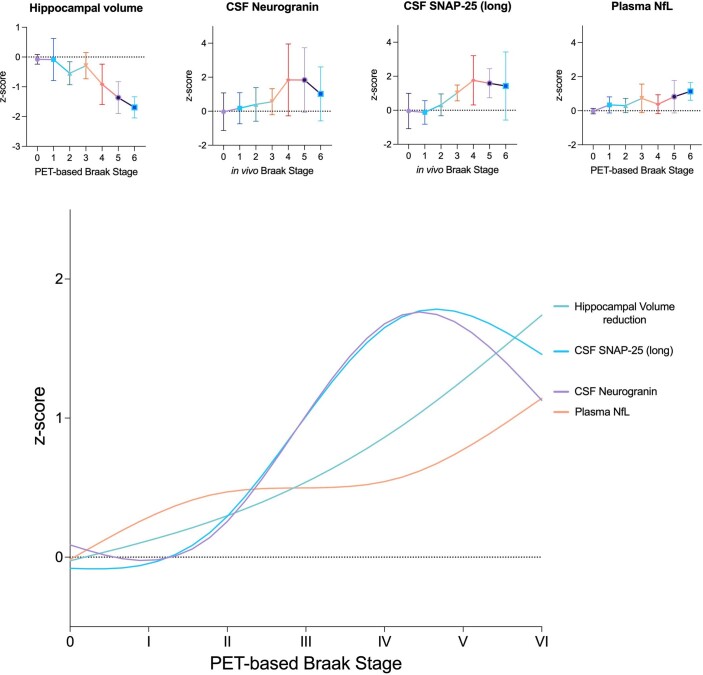
PET-based Braak stage is associated with imaging, CSF and plasma measures of neurodegeneration. **Top:** Individual neurodegeneration biomarkers represented with 95% Confidence Intervals. MRI-measured hippocampal volume (adjusted for intracranial volume) increased in severity starting at stage II onwards (n = 324 individuals). Fluid neurodegeneration biomarkers increase with advancing PET-based Braak stage. CSF measurements of SNAP-25 (long) and neurogranin increased at stages III–IV (189 individuals with CSF measurements). No differences were observed between stages 0, I and II. Substantial within-stage variability was observed for plasma NfL (n = 292 individuals). No differences were observed between stages 0, I and II. Both fluid and imaging neurodegeneration biomarkers showed substantial within-stage variability, potentially reflecting the fact that neurodegeneration is a multifactorial process, influenced by factors other than tau-mediated neurodegeneration. **Bottom:** Neurodegeneration biomarker curves superimposed according to PET-based Braak stage. Note that Hippocampal volume was inverted in order to compare the degree of abnormality.

Figure 4 represents associations between multiple domain-specific and global cognitive outcomes with respect to PET-based Braak stage. Individuals categorized as PET-based Braak stage II had memory dysfunction in the absence of dysfunction in executive, language or visuospatial domains at the group level, compatible with tau accumulation restricted to medial temporal regions. Differences in summary cognitive measures such as the MMSE and Montreal Cognitive Assessment (MOCA) were observed by PET-based Braak stage IV. When assessing the relationship between PET-based Braak stage and clinical dementia severity as assessed by the Clinical Dementia Rating (CDR), we observed that stages 0–II were compatible with the absence of dementia (CDR = 0), whereas most individuals at Braak stages III–IV had a CDR of 0.5, indicating very mild dementia. PET-based Braak stages V and VI were incompatible with normal cognition, with most individuals at Braak stage VI having a CDR of 1 or 2. In a subsample of individuals with *PSEN1* mutations (*n* = 14 mutations carriers, total of 21 tau-PET scans including follow-ups), PET-based Braak stage was associated with the estimated years to onset of symptoms (*P* < 0.0001). Summary statistics for all cognitive outcome comparisons are provided in Supplementary Table 6. Associations between PET-based Braak stage and neurodegeneration biomarkers (hippocampal volume, CSF biomarkers of pre- and postsynaptic integrity, and plasma neurofilament light) are reported in the Extended Data Fig. 5.

## Discussion

Using high-affinity tau imaging, we applied the Braak histopathological staging system for AD to living individuals. PET-based Braak stage had stage-specific associations with amyloid-PET abnormality, several fluid measures of pTau species, and was closely associated with the severity of dementia. The extension of a histopathological staging system to living individuals contributes to the emerging biological research framework for AD^5^ by providing a framework to assess AD severity in living humans.

Using PET-based Braak staging provides an opportunity to study the dynamics of AD biomarker changes in living individuals. CSF measures of pTau at four different phosphorylation sites evolved differentially with respect to PET-based Braak stage. Tau-PET abnormality in early Braak regions was associated with modest increases in CSF pTau_235_, pTau_231_, pTau_217_ and pTau_181_ that were detectable at PET-based Braak stage II, whereas no CSF differences were detectable at stage I, possibly due to the relatively restricted distribution and magnitude of tau at these stages. At stage II, CSF concentrations of pTau_231_ had the largest differences with stage 0 of any tau phosphorylation site, in line with recent data from the ALFA cohort reporting abnormality in pTau_231_ before other phosphorylation sites^15^. Starting at stage III, larger increases in CSF pTau_235_, pTau_231_, pTau_217_ and pTau_181_ were observed, with CSF pTau_181_ demonstrating the largest increases. The dramatic change of CSF concentrations of pTau around Braak stage III and onward may be related to the expansion of tau pathology outside the medial temporal lobe characteristic of these stages^8–10^. At late Braak stages, we observed a plateau of CSF pTau concentrations. The continued aggregation of tau neurofibrillary tangles at later stages despite similar levels of CSF pTau abnormality (which reflect a pathological state at a specific time point) highlights the advantages of PET for disease staging. The order of CSF pTau species abnormality are largely in line with recent studies of two pTau phosphorylation signatures in autosomal dominant AD^16^ (181 and 217), in which abnormality in 217 and 181 are detectable before other species. Our results are in agreement with recent reports suggesting that CSF measures of pTau_181_ and pTau_217_ increase before widespread neocortical tau-PET abnormality^16,17^. However, our study also provides evidence that CSF pTau biomarker concentrations increase in relation to isolated medial temporal tau pathology. Overall, these results suggest that the topographical information provided by high-affinity tau-PET imaging can provide important information about detectable early changes associated with AD and their evolution with increasing disease severity.

When investigating associations between amyloid-PET accumulation and PET-based Braak stage, we made several interesting findings. First, PET-based Braak stages I and II were compatible with both amyloid-PET positivity and negativity, supporting the notion that tau accumulation in early Braak regions can be associated with other factors such as aging. Second, PET-based Braak stage III and above occurred almost exclusively in the presence of amyloid-PET positivity, supporting models of AD in which amyloid-β is required for the propagation of tau pathology across the neocortex^18^. This notion is further corroborated by the substantial increases in the magnitude of fluid pTau species which also occurred at stages III–IV. Third, a plateau of amyloid-β accumulation was observed at PET-based Braak stage IV. The relative stability of amyloid-PET levels from stages IV–VI may be important information for the dosing of anti-amyloid therapeutic agents in trials and clinical settings.

PET-based Braak stage was associated with cognitive dysfunction and severity of dementia. Braak stage II was associated with isolated memory impairment in the absence of deficits in executive, language or visuospatial domains, compatible with tau accumulation restricted to medial temporal regions. Furthermore, early Braak stages evolved in the absence of detectable global cognitive impairment with summary cognition measures such as the MMSE or MOCA. When assessing the relationship between CDR and PET-based Braak stage, we observed that Braak stages 0, I and II were compatible with normal cognition. Braak stage III and IV were most frequently associated with a CDR of 0.5, commonly observed in individuals with MCI^19^. Some individuals at PET-based Braak stage V had a CDR of 1, indicating mild dementia, whereas Braak stage VI was associated with mild and moderate dementia. In the subsample of individuals with autosomal dominant AD, PET-based Braak stage increased sequentially leading up to the estimated onset of symptoms. Although these results require replication in larger cohorts, symptom onset took place approximately between Braak stages II and IV, with increasing PET-based Braak stage associated with the continued worsening of cognitive impairment.

**Fig. 5:**
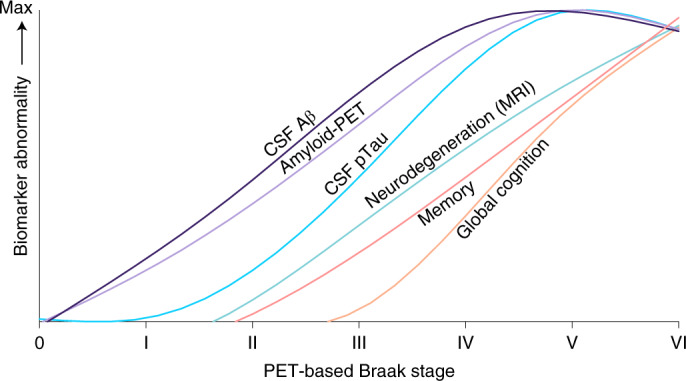
AD biomarker abnormalities in relation to the topography of cerebral tau pathology. Summary of data-driven AD biomarker abnormalities from the perspective of PET-based Braak staging. Trajectories of scaled biomarker data are fitted with LOESS regression. In this conceptual framework, the *x* axis represents Braak stage and not time. Therefore, unlike other AD biomarker models, the *x* axis is not indented to represent the linear temporal evolution of AD and instead displays multiple AD pathophysiological changes in relation to the spatial distribution of tau pathology measured with tau-PET. Similar to other models, biomarker curves represent group-level biomarker changes, and individual-level variability is expected (i.e., Braak stages I and II were compatible with both amyloid-β positivity and negativity at the individual level). However, even in the tau-centric framework, detectable CSF and PET continuous measures of amyloid-β preceded elevated pTau concentrations measured in CSF. CSF pTau abnormality accelerated dramatically between Braak stages III and VI. Neurodegeneration indexed by hippocampal volume was closely followed by memory dysfunction. Dysfunction in global cognition rose slightly around stages III–VI and more dramatically at stages V–VI. PET-based Braak staging also provides a framework for testing new biomarkers.

PET-based Braak staging provides a framework to model changes in AD biomarkers that may complement models from autosomal dominant AD^3,16^, Down syndrome^20^ and temporal models from sporadic AD^2^. An example of a dynamic AD biomarker framework using PET-based Braak staging is provided in Fig. 5. Similar to existing biomarker frameworks for AD^2,3,16,17,20^, PET-based Braak staging provides the opportunity to evaluate the course of abnormality of various biomarkers over the natural history of AD. Unique features of a model based on Braak stages include (1) modeling the disease from the perspective of the evolution of tau pathology and (2) the correspondence with existing gold-standard histopathological frameworks for AD diagnosis^6,7^. PET-based Braak staging using tau-PET also provides a framework for the validation of new AD biomarkers as they become available^12^. Despite some advantages, it is important to emphasize that biomarker modeling of AD using PET-based Braak staging may be less well positioned to detect and model very early AD changes that are considered to precede tau aggregation, such amyloid-β accumulation^21^.

Advances in molecular imaging are anticipated to refine existing diagnostic classification schemes for AD^14^. Several studies have reported that the topography of tau-PET signal at the group level largely conforms to the histopathological work reported by Braak and Braak^22–25^. However, the present study instead used individual-level tau-PET data to assign a Braak stage to each study participant, subsequently using individual-level staging information to model biomarker changes and assess relationships to cognitive impairment. Biomarker-based measurements of disease severity have several important advantages over clinical assessment of symptoms. Because of AD’s long preclinical phase in which amyloid-β and tau aggregate in the absence of symptoms^2–4^, biomarker-based disease staging can identify early changes in AD long before they can be reliably captured by cognitive decline. Moreover, cognitive testing outcomes are subject to ceiling effects, floor effects and practice effects, all of which are complicated by cognitive reserve^26^. Finally, the specificity of tau-PET for the 3 R + 4 R tau that characterizes AD allows for the estimation of AD severity specifically, in contrast to cognitive decline, which is often the result of multiple comorbidities^27^. Braak staging also provides a framework for tracking the spatial progression of neurofibrillary tangle changes in individuals who are considered AD biomarker negative. Specifically, examining regions of early tau aggregation (e.g., in stages I and II) with tau-PET may provide higher sensitivity for detecting tau aggregation in asymptomatic individuals than with commonly employed meta-ROIs, which have high specificity for a diagnosis of AD dementia^28^ but may have lower sensitivity for detecting isolated medial temporal tau. Furthermore, PET-based Braak staging provides a framework for the identification of biomarker changes at specific points in the disease course that may be less easily identified based on summary continuous measures of tau-PET. Similar to other staging systems in medicine, is important to emphasize that in vivo disease staging in AD is not intended to replace assessment of symptoms, but rather can be used in tandem to provide complementary information about the disease course, especially at early stages^29^. Furthermore, in vivo staging of AD severity does not imply the need to reject biomarker dichotomization for other purposes. Biomarker dichotomization provides useful information about clinical diagnostic accuracy^30^, changes in patient management^31^ and AD epidemiology^32^. Biomarker dichotomization is also helpful in situations where numerous biomarkers are being evaluated concurrently (i.e., A/T/(N) classification)^33^. Finally, the tau staging system proposed by Braak and Braak does not capture the full scope of pathological changes in AD; biomarkers of amyloid-β are required to monitor the evolution of early AD pathological change^5^.

It is important to draw a conceptual distinction between models of tau staging^8,9^ and experimental models of tau propagation^34–36^. Braak staging is a histopathological construct that allows for the differentiation of several disease phases based on the anatomical distribution of tau neurofibrillary tangles^11^. This staging system is part of the gold standard for AD diagnosis, in which Braak stage III/IV and above accompanied by significant amyloid-β plaques and neuritic plaques is considered to be a sufficient explanation for dementia^6,7^. In contrast, models of tau propagation test hypotheses related to changes in the anatomical distribution of tau pathology over time. Although recent tau-PET studies have provided evidence for variability in tau propagation patterns^34,35^, transneuronal tau propagation models are not mutually exclusive with a Braak staging scheme^36,37^. Indeed, variability in regional tau pathology between individuals at the same Braak stage is described in the Braak staging model^8–10^. Furthermore, histopathological studies of atypical AD phenotypes suggest that the Braak staging model can be meaningfully applied despite regional differences in the magnitude of neurofibrillary tangles that characterize different focal cortical syndromes^38,39^. Therefore, models of tau propagation likely provide complementary information to Braak stage regarding an individual’s future tau accumulation^34,35^.

Our study has limitations. The most important limitation of our study is the paucity of case-to-autopsy studies conducted in individuals with an antemortem [^18^F]MK6240 scan. Although recent work suggests agreement between PET-based Braak stage from [^18^F]MK6240 and Braak stage at autopsy^40^, larger studies in more diverse populations are needed to increase confidence in this finding. Case-to-autopsy studies have suggested that [^18^F]Flortaucipir PET cannot reliably detect the presence of tau neurofibrillary tangles in early Braak regions at autopsy^41,42^. It is unknown to what extent the fivefold-higher B_max_/*K*_d_ (concentration of available binding sites/equilibrium dissociation constant) ratio of [^18^F]MK6240 for neurofibrillary tangles^43^ will result in stronger correlations with histopathological findings than those reported with [^18^F]Flortaucipir. The limited spatial resolution of PET imaging also poses limitations regarding the in vivo detection of tau neurofibrillary tangles, particularly in small regions of the medial temporal lobe. Despite employing nonoverlapping ROIs, the anatomical proximity of the transentorhinal and entorhinal cortices may result in suboptimal ability to detect differences in tau accumulation between these regions. Furthermore, the histopathological Braak staging framework is based on staining procedures that preferentially identify specific tau phosphorylation sites^44^; the degree to which these phosphorylation sites contribute to tau-PET ligand binding signal is unknown. It is also presently unclear to what extent different fluid pTau biomarkers reflect cerebral amyloid-β or tau neurofibrillary tangle pathology. Another limitation of our study is that all disease staging models are inherently artificial constructs; attempts to divide continuous processes into discrete stages will always be met with issues of sensitivity and specificity^11^. This issue is similar to dichotomization of continuous biomarkers, although the use of several stages may attenuate the risks associated with positive/negative dichotomization. Future work is needed develop PET-based Braak staging methods for other tau-PET radioligands. Future studies should also address whether copathologies influence the AD biomarker trajectories described in this paper. An advantage of our study is the use of a tau-PET ligand with subnanomolar affinity for tau neurofibrillary tangles^43^, resulting in high sensitivity to detect early tau aggregation.

In conclusion, our results support PET-based Braak staging as a framework to model the natural history of AD and monitor AD severity in living humans. Braak staging may also be useful for determining inclusion in therapeutic trials^45,46^.

## Methods

### Participants

We assessed 179 CU older adults, 80 individuals with MCI and 65 individuals with Alzheimer’s clinical syndrome enrolled in the Translational Biomarkers of Aging and Dementia (TRIAD) cohort^47^ who had amyloid-β-PET with [^18^F]AZD4694, tau-PET with [^18^F]MK6240 and magnetic resonance imaging (MRI) at baseline. A subsample of 140 individuals had a follow-up [^18^F]MK6240 after one year, and 84 who had a follow-up [^18^F]MK6240 after 2 years (65 individuals had [^18^F]MK6240 PET scans at baseline, 1 year and 2 years). Participants had a detailed clinical and cognitive assessment, including the Clinical Dementia Rating (CDR) and Mini-Mental State Examination (MMSE) and neuropsychological testing. CU individuals had no objective cognitive impairment, a CDR score of 0, and were asked to report any subjective cognitive decline in a questionnaire given during screening. Individuals with MCI had subjective and/or objective cognitive impairment, relatively preserved activities of daily living, and a CDR score of 0.5. Mild-to-moderate Alzheimer’s clinical syndrome patients with dementia had a CDR score between 0.5 and 2 and met the NIA-AA criteria for probable AD determined by a dementia specialist^48^. The Alzheimer’s clinical syndrome group included individuals with nonamnestic phenotypes, including primary progressive aphasia^49^, posterior cortical atrophy^50^ and behavioral/dysexecutive AD^51^. Participants were excluded from the present study if they had neurological, psychiatric or systemic conditions that were not adequately controlled through a stable medication regimen. Other exclusion criteria were active substance abuse, recent head trauma, recent major surgery or MRI/PET safety contraindications. The study was approved by the Montreal Neurological Institute PET working committee and the Douglas Mental Health University Institute Research Ethics Board. Written informed consent was obtained for all participants.

### PET processing

Study participants had a T1-weighted MRI, and [^18^F]AZD4694 PET and [^18^F]MK6240 PET scans were acquired using a brain-dedicated Siemens high-resolution research tomograph. [^18^F]MK6240 PET images were acquired at 90–110 min after the intravenous bolus injection of the radiotracer and reconstructed using an ordered subset expectation maximization algorithm on a 4D volume with four frames (4 × 300 s), as previously described^52^. [^18^F]AZD4694 PET images were acquired at 40–70 min after the intravenous bolus injection of the radiotracer and reconstructed with the same ordered subset expectation maximization algorithm on a 4D volume with three frames (3 × 600 s)^53^. A 6-min transmission scan with a rotating ^137^Cs point source was conducted at the end of each PET acquisition for attenuation correction. Images were corrected for motion, decay, dead time and random and scattered coincidences. Briefly, PET images were automatically registered to T1-weighted image space, and the T1-weighted images were linearly and nonlinearly registered to the Montreal Neurological Institute (MNI) reference space. To minimize the influence of meningeal spillover into adjacent brain regions, [^18^F]MK6240 images were meninges-striped in native space before transformations and blurring^25^. PET images were linearly and nonlinearly registered to the MNI space using the transformations from the T1-weighted image to MNI space and from the PET image to T1-weighted image space. [^18^F]MK6240 SUVRs were calculated using the cerebellar crus I gray matter as a reference region^25,54^, as derived from the SUIT cerebellum atlas^55^. [^18^F]AZD4694 SUVRs were calculated using the whole cerebellum gray matter as the reference region^53^. PET images were spatially smoothed to achieve an 8-mm full-width at half-maximum resolution. The global [^18^F]AZD4694 SUVR composite included the precuneus, prefrontal, orbitofrontal, parietal, temporal and cingulate cortices^53,56^. [^18^F]AZD4694 SUVR positivity was determined as an SUVR > 1.55, as detailed elsewhere^53^.

### MRI acquisition and processing

Structural MRI data were acquired at the MNI for all participants on a 3 T Siemens Magnetom scanner using a standard head coil. Hippocampal volume was assessed using FreeSurfer version 6.0 using the Desikian–Killiany–Touriner atlas gray matter segmentation. Hippocampal volume was adjusted for intracranial volume.

### PET-based Braak staging methods

The transentorhinal cortex was segmented in the stereotaxic space on 1-mm isotropic voxels^25^ using a validated MRI segmentation technique and identifiable anatomical landmarks^57,58^. The transentorhinal ROI was segmented in the medial bank of the rhinal sulcus, which corresponds to the transition area between the entorhinal and perirhinal cortices^59,60^. The transentorhinal and entorhinal cortex ROIs did not overlap with each other. Furthermore, the peaks of the transentorhinal and entorhinal cortex ROI probabilistic distributions were 14 mm apart, which is just under two full-width at half-maximum of the PET resolution in this study (8 mm). Despite this, the outer edges of the transentorhinal and entorhinal cortex ROIs were as close as 2 mm apart in some slices. For these reasons, it is possible that the distinction between PET-based stages I and II is driven by [^18^F]MK6240 uptake in the hippocampus.

**Extended Data Fig. 6:**
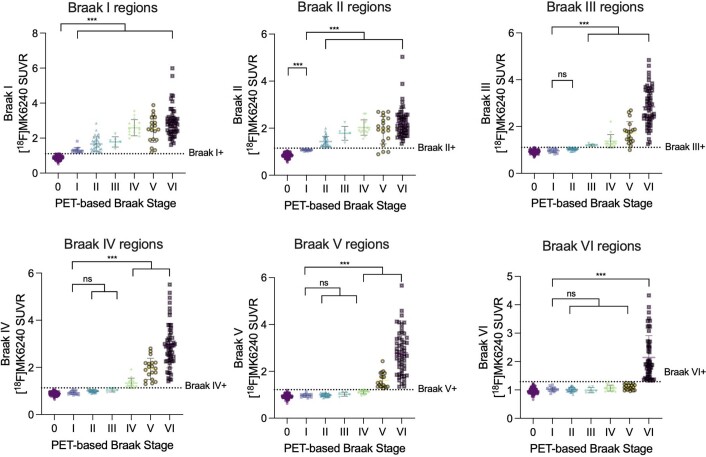
Full distribution of [^18^F]MK6240 SUVR in Braak regions. Group means are represented by lines, error bars indicate standard deviation. The three individuals with focal cortical syndromes who were Braak stage discordant were not included in this figure. ***: p < 0.001; two-sided ANOVA corrected for multiple comparisons using Dunnett’s correction (n = 324 individuals).

Tau-PET Braak stage segmentation was previously described elsewhere^25,40,61^. Stages consisted of the following regions: Braak I (transentorhinal), Braak II (entorhinal and hippocampus), Braak III (amygdala, parahippocampal gyrus, fusiform gyrus and lingual gyrus), Braak IV (insula, inferior temporal, lateral temporal, posterior cingulate and inferior parietal), Braak V (orbitofrontal, superior temporal, inferior frontal, cuneus, anterior cingulate, supramarginal gyrus, lateral occipital, precuneus, superior parietal, superior frontal and rostromedial frontal) and Braak VI (paracentral, postcentral, precentral and pericalcarine)^8–10^. The Desikan–Killiany–Tourville atlas was used to define the ROIs for the PET Braak-like stages^62^. In addition to Braak stages, we assessed [^18^F]MK6240 SUVR in the temporal meta-ROI, a commonly employed summary measure of tau-PET^28,56^. Temporal meta-ROI tau-PET positivity was determined using a previously validated threshold^30^. A baseline Braak stage was defined for each subject corresponding to the latest stage where tau-PET abnormality was detected. Tau-PET abnormality in Braak regions was defined using cutoffs determined as 2.5 s.d. higher than the mean SUVR of CU young adults as previously described^25,40^. Similar cutoffs were observed when using Gaussian mixture modeling^25^. Individual data points for all tau-PET data according to PET-based Braak stage are presented in Extended Data Fig. 6.

To assess longitudinal stability of PET-based Braak stage, we examined 159 individuals who had a follow-up [^18^F]MK6240 at either 1 year (*n* = 140) or 2 years after baseline (*n* = 83). The goal of these analyses was to test the hypothesis that individuals should either remain at the same PET-based Braak stage over time or progress to the next Braak stage. Decreases in Braak stage over time (i.e., stage III at baseline, stage II or lower at follow-up) would suggest instability of the staging system. On the other hand, sequential increases in stage over time or within-stage stability at follow-up is compatible with a hierarchical Braak staging model of tau progression. Demographics of the subsample with longitudinal tau-PET are presented in Supplementary Table 7.

### Neuropsychological testing

**Extended Data Fig. 7:**
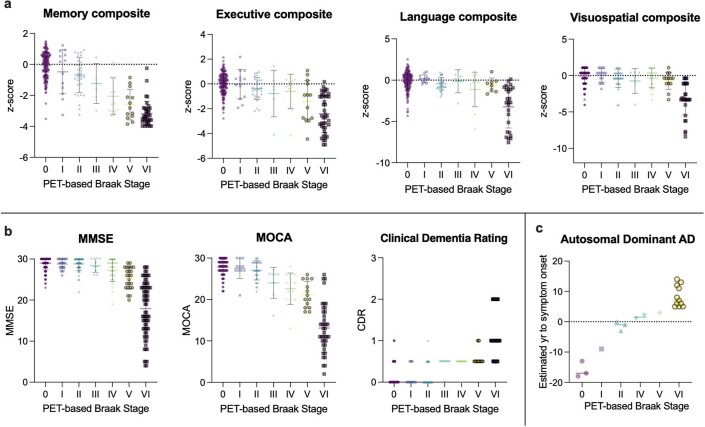
Full distributions of Cognitive outcomes grouped by Braak stage. Group means are represented by lines, error bars indicate standard deviation where applicable. a: n = 292 individuals with neuropsychological assessments available. b: n = 324 individuals with summary cognitive assessments available. c: n = 14 PSEN1 mutation carriers; total of 21 scans including follow-ups.

Neuropsychological evaluation consisted of memory, language and visuospatial and executive cognitive domains. Memory was assessed using the immediate and delayed logical memory, as well as the Rey auditory verbal learning immediate and delayed recall. Language was assessed with the category fluency and Boston naming tests. Visuospatial function was assessed with the Birmingham object recognition battery. Executive function was assessed using trail-making test–B time, digit span backward and letter fluency (D words). Raw test scores were *Z* transformed using mean and s.d. values from the CU older adults. *Z* scores were averaged across all tests within each cognitive domain, resulting in a composite score for each domain. Individual data points for neuropsychological and summary cognitive testing outcomes according to PET-based Braak stage are reported in Extended Data Fig. 7.

### Fluid biomarkers

**Extended Data Fig. 8:**
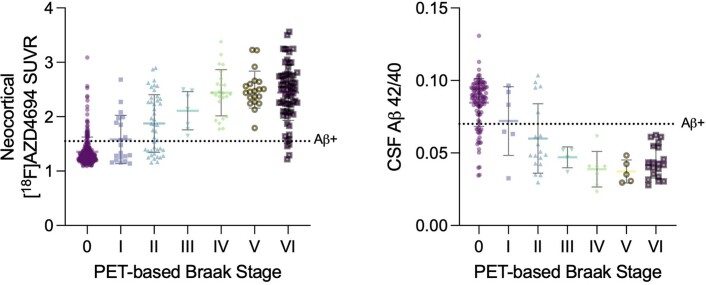
Full distribution of [^18^F]AZD4694 SUVR and CSF Aβ 42/40 grouped by Braak stage. Group means are represented by lines, error bars indicate standard deviation. Note that the CSF biomarkers represent a subsample of individuals (n = 189 individuals with CSF evaluations) which did not include the amyloid-PET negative individuals at Braak stage VI (n = 324 individuals with amyloid-PET).

CSF samples were collected with lumbar puncture under local anesthesia using an 18-G ‘introducer’ to penetrate the interspinous ligaments, followed by dural puncture using the 24-G Sprotte atraumatic needle. 29 ml fluid was collected with polypropylene syringes, from which the initial 4 ml was sent to local laboratory for routine analyses. The remaining volume was transferred to polypropylene tubes and centrifuged at 20 °C for 10 min at 2,200 *g*. Samples were then rapidly frozen for permanent storage at −80 °C. We cross-sectionally assessed several pTau epitopes measured from CSF in a subsample of 189 individuals: pTau_181_, pTau_217_, pTau_231_ and pTau_235_. CSF concentrations of pTau_181_ were assessed with the LUMIPULSE G1200 instrument from Fujirebio, whereas CSF pTau_217_ was quantified using a custom single molecule array (Simoa) assay, and CSF pTau_231_ was quantified using an enzyme-linked immunosorbent assay^15^. CSF concentrations of pTau_235_ were quantified using an in-house-developed Simoa assay comprising a rabbit polyclonal antibody specific for pTau_235_ conjugated to paramagnetic beads and mouse monoclonal Tau12 (epitope 6–18 aa) as a detector^63^. CSF concentrations of amyloid-β (Aβ40 and Aβ42) were assessed using the fully-automated LUMIPULSE G1200 instrument (Fujirebio). LUMIPULSE measured the 42-aa-long form of amyloid-β (Aβ42) and the 40-aa-long form of amyloid-β (Aβ40) using antibody-coated beads for capture, as well as monoclonal antibodies for detection^64^. For amyloid-β analyses using CSF measurements, we used the Aβ42/Aβ40 ratio (Aβ42 concentrations normalized to concentrations of Aβ40), as a recent review provides substantial evidence that the Aβ42/Aβ40 ratio has superior diagnostic performance (lower false-positive and lower false-negative rates)^65^. Amyloid-β positivity on CSF was determined based on a published cutoff of a Aβ42/Aβ40 ratio of 0.068 from the LUMIPULSE assay^64^. Individual data points for CSF Aβ42/Aβ40 ratio are reported in Extended Data Fig. 8. CSF biomarkers of the dendritic protein Neurogranin were quantified using an in-house assay at the Clinical Neurochemistry Laboratory, University of Gothenburg by scientists blinded to participant clinical information. CSF concentrations of SNAP-25, a biomarker of synaptic degeneration, were quantified using immunoprecipitation mass spectrometry.

**Extended Data Fig. 9:**
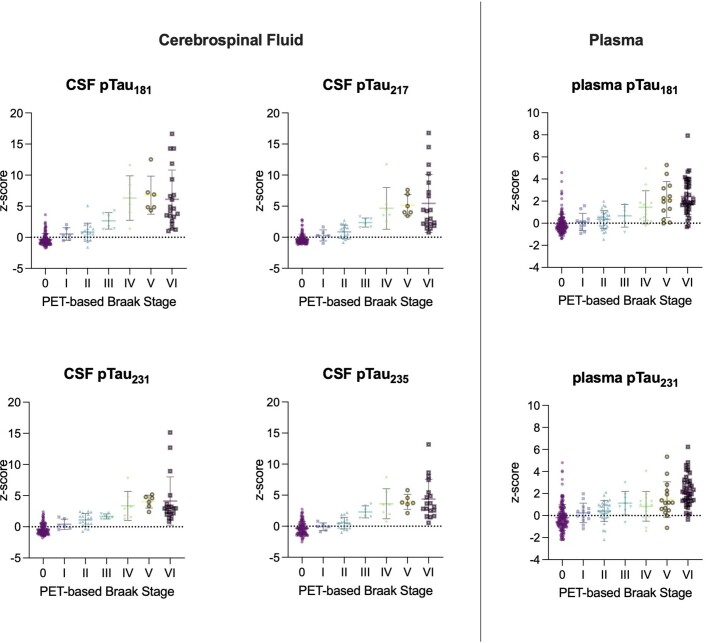
Full distribution of fluid phosphorylated tau concentrations grouped by Braak stage. Group means are represented by lines, error bars indicate standard deviation (n = 189).

Blood samples were collected following previously described protocols^66^. Plasma pTau_181_ and plasma pTau_231_ were measured in the Clinical Neurochemistry Laboratory, University of Gothenburg by scientists blinded to participant clinical information. All plasma pTau biomarkers were measured using an in-house Single Molecular Array (Simoa) method (Simoa HD-X instruments, Quanterix)^66,67^. Plasma neurofilament light concentrations were measured using an in-house Simoa assay in the Clinical Neurochemistry Laboratory, University of Gothenburg as previously described^68^. Individual data points for all fluid pTau concentrations are reported in Extended Data Fig. 9.

### Estimated years to the onset of symptoms for autosomal dominant AD subsample

Due to the relatively consistent age of symptom onset for each autosomal dominant AD-causing mutation, a measure of the expected onset of symptoms can be estimated based on family history^3,16^. Using a semistructured interview, the age at which symptoms appeared in the parent was established. Medical records and consultation with other family members was performed when necessary. The parental age of onset was subtracted from the age of the participants in order to create an estimation of the years to the onset of symptoms.

### Statistical methods

Demographic characteristics were evaluated using *t* tests and *χ*^2^ analyses performed in R version 3.5.3. We calculated mean images of [^18^F]MK6240 SUVR and [^18^F]AZD4694 SUVR according to PET-based Braak stage. To represent biomarker trajectories on a common scale while also permitting the visualization of different magnitudes of biomarker changes, all biomarkers were standardized using the mean and s.d. from the CU older adults (irrespective of their Braak stage). Domain-specific cognitive test scores were also standardized using mean and s.d. of the CU older adults. Imaging, CSF and plasma biomarkers were compared between PET-based Braak stages using ANOVA. ANOVA model assumptions were tested using Anderson–Darling test for normality and Bartlett’s test for equal s.d. values. Because criteria for equal variances were not met, we employed Brown–Forsythe ANOVA. Post-hoc tests were conducted with Dunnett’s correction for multiple comparisons^69^. Cognitive composite *Z* scores were also assessed between PET-based Braak stages using Brown–Forsythe ANOVA. To compare different biomarkers with respect to their overall maximal levels of abnormality^2,16,17^, we scaled all biomarker *Z* scores to a common scale of 0–10, with 10 representing maximum abnormality. These curves were fit using LOESS regression.

### Reporting Summary

Further information on research design is available in the Nature Research Reporting Summary linked to this article.

## Supplementary information

Supplementary Information Supplementary Tables 1–7.

Reporting Summary

## Figures and Tables

**Extended Data Fig. 4:**
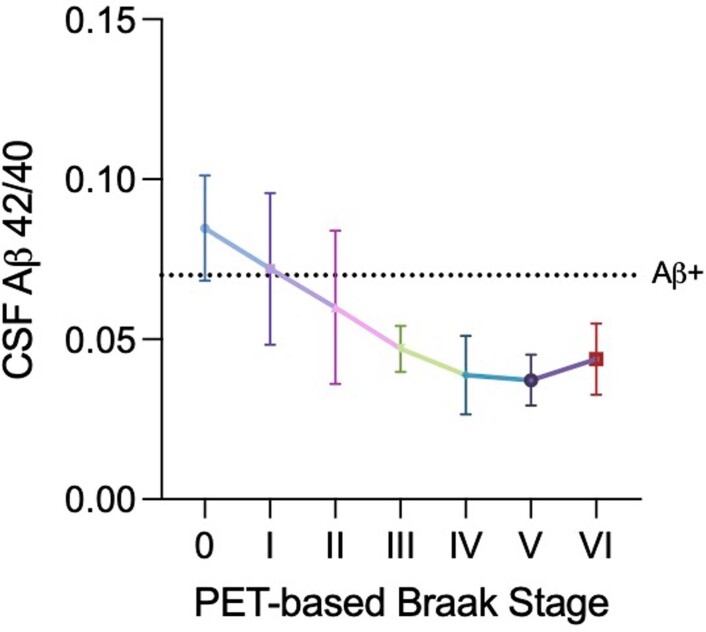
Braak stages evolve with respect to CSF amyloid-β concentrations. Means are represented by shapes and error bars indicate standard deviation. Dashed line represents a previously validated threshold for amyloid positivity. Similar to amyloid-PET data, most individuals at Braak stage 0 were amyloid negative. PET-based Braak stages I and II were compatible with both amyloid positivity and negativity. A higher proportion of individuals were CSF amyloid-positive at stage II and were amyloid-PET positive at stage II. A plateau of amyloid abnormality was observed at late Braak stages. 189 individuals had CSF measurements available.

## Data Availability

All requests for raw and analyzed data and materials will be promptly reviewed by McGill University to verify if the request is subject to any intellectual property or confidentiality obligations. Anonymized data will be shared upon request to the study’s senior author from a qualified academic investigator for sole the purpose of replicating the procedures and results presented in this article. Any data and materials that can be shared will be released via a material transfer agreement. Data are not publicly available due to information that could compromise the privacy of research participants. Related documents, including study protocol and informed consent forms, can similarly be made available upon request.
