# Supplementary material for: Biomarker modeling of Alzheimer’s disease using PET-based Braak staging
Source: Nat Aging. 2022 Apr 25;2(6):526–35. doi: 10.1038/s43587-022-00204-0 (PMC10154209; doi:10.1038/s43587-022-00204-0)
Supplement: Supplementary file 1 — Supplementary Tables 1–7. [file 43587_2022_204_MOESM1_ESM.pdf]

nature portfolio

https://doi.org/10.1038/s43587-022-00204-0

Supplementary information

Biomarker modeling of Alzheimer's disease using PET-based Braak staging

In the format provided by the authors and unedited

Supplemental Table 1. Summary of statistical comparisons of regional tau-PET SUVRs across individuals at different *in vivo* Braak stages

**Braak I Regions**	**Mean difference**	**95% CI of difference**	**Adjusted *p* value**
**0 vs I**	-0.424	-0.564 to -0.283	<0.0001
**I vs II**	-0.776	-0.991 to -0.559	<0.0001
**I vs III**	-0.897	-1.465 to -0.329	<0.0001
**I vs IV**	-1.707	-2.115 to -1.299	<0.0001
**I vs V**	-1.823	-2.360 to -1.286	<0.0001
**I vs VI**	-2.020	-2.398 to -1.643	<0.0001

**Braak II Regions**	**Mean difference**	**95% CI of difference**	**Adjusted *p* value**
**0 vs I**	0.244	0.171 to 0.317	<0.0001
**I vs II**	-0.322	-0.476 to -0.166	<0.0001
**I vs III**	-0.693	-1.268 to -0.118	<0.0001
**I vs IV**	-0.936	-1.224 to -0.649	<0.0001
**I vs V**	-1.011	-1.381 to -0.640	<0.0001
**I vs VI**	-1.137	-1.393 to -0.880	<0.0001

**Braak III Regions**	**Mean difference**	**95% CI of difference**	**Adjusted *p* value**
**0 vs I**	0.048	-0.026 to 0.121	0.2428
**I vs II**	-0.082	-0.157 to -0.006	0.0141
**I vs III**	-0.243	-0.333 to -0.152	<0.0001
**I vs IV**	-0.409	-0.662 to -0.154	<0.0001
**I vs V**	-0.832	-1.227 to -0.436	<0.0001
**I vs VI**	-1.878	-2.215 to -1.540	<0.0001

**Braak IV Regions**	**Mean difference**	**95% CI of difference**	**Adjusted *p* value**
**0 vs I**	0.039	-0.030 to 0.110	0.3667
**I vs II**	-0.076	-0.148 to -0.003	0.0173
**I vs III**	-0.121	-0.284 to 0.041	0.1008
**I vs IV**	-0.407	-0.595 to -0.219	<0.0001
**I vs V**	-1.102	-1.457 to -0.745	<0.0001
**I vs VI**	-1.996	-2.381 to -1.612	<0.0001

**Braak V Regions**	**Mean difference**	**95% CI of difference**	**Adjusted *p* value**
**0 vs I**	0.041	-0.023 to 0.105	0.2618
**I vs II**	-0.020	-0.088 to 0.048	0.9268
**I vs III**	-0.051	-0.204 to 0.102	0.7348
**I vs IV**	-0.152	-0.241 to -0.062	<0.0001
**I vs V**	-0.681	-0.967 to -0.395	<0.0001
**I vs VI**	-1.820	-2.193 to -1.446	<0.0001

**Braak VI Regions**	**Mean difference**	**95% CI of difference**	**Adjusted *p* value**
**0 vs I**	0.058	-0.013 to 0.128	0.0872
**I vs II**	0.010	-0.065 to 0.086	0.9985
**I vs III**	0.034	-0.133 to 0.201	0.9579
**I vs IV**	-0.037	-0.143 to 0.068	0.8421
**I vs V**	-0.073	-0.167 to 0.020	0.1211
**I vs VI**	-1.114	-1.431 to -0.7977	<0.0001

All analyses conducted with ANOVA (Brown-Forsythe) test. All ANOVAs significant at $p < 0.001$. Adjusted p values reflect p values after multiple comparisons correction with Dunnett's T3 multiple comparisons test.

Supplementary Table 2. Summary of Amyloid-PET statistical comparisons between Braak stages

**Amyloid-PET SUVR**	**Mean difference**	**95% CI of difference**	**Adjusted *p* value**
**0 vs I**	-0.228	-0.550 to 0.095	0.1810
**0 vs II**	-0.520	-0.766 to -0.274	<0.0001
**0 vs III**	-0.753	-1.334 to -0.172	0.0071
**0 vs IV**	-1.085	-1.362 to -0.807	<0.0001
**0 vs V**	-1.143	-1.391 to -0.894	<0.0001
**0 vs VI**	-1.082	-1.271 to -0.892	<0.0001
**IV vs V**	-0.058	-0.375 to 0.259	0.9417
**IV vs VI**	0.0031	-0.289 to 0.295	>0.9999
**V vs VI**	0.061	-0.207 to 0.329	0.8964

Supplementary Table 3. Summary of CSF Aβ42/Aβ40 ratio statistical comparisons between Braak stages

**CSF Aβ42/Aβ40 ratio**	**Mean difference**	**95% CI of difference**	**Adjusted *p* value**
**0 vs I**	-0.119	-1.731 to 1.494	0.9992
**0 vs II**	-1.073	-1.989 to -0.157	0.0069
**0 vs III**	-1.692	-2.624 to -0.7603	0.0018
**0 vs IV**	-2.084	-2.991 to -1.178	<0.0001
**0 vs V**	-2.163	-2.961 to -1.365	<0.0001
**0 vs VI**	-1.849	-2.314 to -1.384	<0.0001
**IV vs V**	-0.078	-0.986 to 0.830	0.9887
**IV vs VI**	0.235	-0.581 to 1.052	0.7310
**V vs VI**	0.313	-0.400 to 1.028	0.4016

Supplemental Table 4. Percentage of amyloid-positive individuals according to PET and CSF modalities at each Braak stage in the cross-sectional cohort

PET-Based Braak Stage	% Amyloid-PET +	% CSF Aβ +
**0**	17%	25%
**I**	42%	40%
**II**	64%	72%
**III**	100%	100%
**IV**	100%	100%
**V**	100%	100%
**VI**	96%	100%

The amyloid-negative individuals at stage VI may represent cases of Neurofibrillary Tangle-Predominant Dementia [1], who display high levels of neurofibrillary tangle pathology in the absence of elevated amyloid-beta and present with a phenotype indistinguishable from Alzheimer's clinical syndrome. The amyloid-PET negative Braak stage VI individuals did not have CSF evaluations.

Supplemental Table 5. Summary of statistical comparisons of CSF and plasma phosphorylated tau species across Braak stages

**CSF pTau181**	**Mean difference**	**95% CI of difference**	**Adjusted *p* value**
**0 vs I**	-0.927	-3.615 to 1.760	0.4629
**0 vs II**	-1.191	-2.327 to -0.055	0.0176
**0 vs III**	-3.139	-8.072 to 1.794	0.0447
**0 vs IV**	-6.664	-13.51 to 0.1811	0.0279
**0 vs V**	-7.612	-15.16 to -0.060	0.0243
**0 vs VI**	-6.455	-9.874 to -3.035	<0.0001
**IV vs V**	-0.947	-7.691 to 5.796	0.9514
**IV vs VI**	0.209	-5.515 to 5.934	0.9992
**V vs VI**	1.157	-4.692 to 7.006	0.8808

**CSF pTau217**	**Mean difference**	**95% CI of difference**	**Adjusted *p* value**
**0 vs I**	-0.586	-3.001 to 1.826	0.7277
**0 vs II**	-1.164	-2.015 to -0.312	0.0017
**0 vs III**	-2.768	-5.435 to -0.101	0.0225
**0 vs IV**	-4.943	-10.42 to 0.533	0.0395
**0 vs V**	-5.691	-10.04 to -1.342	0.0095
**0 vs VI**	-5.738	-9.124 to -2.351	0.0002
**IV vs V**	-0.748	-5.764 to 4.269	0.9429
**IV vs VI**	-0.794	-5.767 to 4.178	0.9477
**V vs VI**	-0.049	-3.944 to 3.850	>0.9999

CSF pTau231	Mean difference	95% CI of difference	Adjusted *p* value
**0 vs I**	-0.935	-4.444 to 2.575	0.5099
**0 vs II**	-1.493	-2.308 to -0.678	<0.0001
**0 vs III**	-1.968	-3.097 to -0.839	0.0032
**0 vs IV**	-3.744	-7.547 to 0.058	0.0268
**0 vs V**	-4.279	-6.920 to -1.637	0.0042
**0 vs VI**	-4.521	-7.426 to -1.616	0.0005
**IV vs V**	-0.534	-3.883 to 2.814	0.9315
**IV vs VI**	-0.776	-4.468 to 2.915	0.8982
**V vs VI**	-0.242	-3.167 to 2.683	0.9931

CSF pTau235	Mean difference	95% CI of difference	Adjusted *p* value
**0 vs I**	-0.257	-1.788 to 1.273	0.9498
**0 vs II**	-0.833	-1.594 to -0.071	0.0122
**0 vs III**	-2.657	-5.979 to 0.664	0.0463
**0 vs IV**	-3.953	-7.887 to -0.018	0.0245
**0 vs V**	-4.303	-7.578 to -1.028	0.0093
**0 vs VI**	-4.710	-7.177 to -2.242	<0.0001
**IV vs V**	-0.350	-3.895 to 3.194	0.9832
**IV vs VI**	-0.757	-4.320 to 2.807	0.8890
**V vs VI**	-0.406	-3.267 to 2.454	0.9641

Plasma pTau181	Mean difference	95% CI of difference	Adjusted *p* value
**0 vs I**	-0.111	-0.979 to 0.757	0.9979
**0 vs II**	-0.352	-0.867 to 0.164	0.2452
**0 vs III**	-0.684	-3.190 to 1.822	0.6460
**0 vs IV**	-1.446	-2.832 to -0.060	0.0188
**0 vs V**	-2.147	-3.729 to -0.564	0.0026
**0 vs VI**	-2.431	-3.283 to -1.579	<0.0001
**IV vs V**	-0.701	-2.432 to 1.031	0.5806
**IV vs VI**	-0.984	-2.359 to 0.390	0.1434
**V vs VI**	-0.284	-1.811 to 1.242	0.9296

Plasma pTau231	Mean difference	95% CI of difference	Adjusted *p* value
**0 vs I**	-0.424	-1.293 to 0.446	0.5187
**0 vs II**	-0.599	-1.177 to -0.022	0.0182
**0 vs III**	-1.309	-2.813 to 0.194	0.0502
**0 vs IV**	-1.022	-2.264 to 0.220	0.0799
**0 vs V**	-1.645	-3.064 to -0.226	0.0085
**0 vs VI**	-2.431	-3.154 to -1.708	<0.0001
**IV vs V**	-0.623	-2.180 to 0.933	0.5951
**IV vs VI**	-1.409	-2.624 to -0.194	0.0084
**V vs VI**	-0.785	-2.140 to 0.568	0.2846

All analyses conducted with ANOVA (Brown-Forsythe) test. All ANOVAs significant at $p < 0.001$. Adjusted p values reflect p values after multiple comparisons correction with Dunnett's T3 multiple comparisons test. P-values for differences between stages were considered statistically

significant at $p < 0.01$ after multiple comparisons correction. All CSF pTau species had detectable differences by Braak stage III. CSF pTau181, 217, 231 and 235 were all significantly different from stage 0 by stage II. Plasma pTau231 was significantly different by Braak stage II and plasma pTau181 was significantly different by stage VI.

Supplementary Table 6. Summary of statistical comparisons of cognitive outcomes between Braak stages

**Memory Composite**	**Mean difference**	**95% CI of difference**	**Adjusted *p* value**
**0 vs I**	0.4307	-0.940 to 1.802	0.8521
**0 vs II**	0.6456	-0.002 to 1.293	0.0256
**0 vs III**	1.195	-1.283 to 3.673	0.0880
**0 vs IV**	2.014	0.914 to 3.113	0.0001
**0 vs V**	2.551	1.589 to 3.514	<0.0001
**0 vs VI**	3.099	2.618 to 3.581	<0.0001

**Executive Composite**	**Mean difference**	**95% CI of difference**	**Adjusted *p* value**
**0 vs I**	0.04131	-1.114 to 1.196	>0.9999
**0 vs II**	0.3904	-0.142 to 0.923	0.1717
**0 vs III**	0.8023	-2.802 to 4.406	0.8576
**0 vs IV**	0.6201	-0.634 to 1.875	0.4846
**0 vs V**	1.432	-0.211 to 3.075	0.0557
**0 vs VI**	2.403	1.658 to 3.147	<0.0001

**Language Composite**	**Mean difference**	**95% CI of difference**	**Adjusted *p* value**
**0 vs I**	-0.2798	-0.801 to 0.241	0.4549
**0 vs II**	0.3168	-0.170 to 0.804	0.2950
**0 vs III**	0.01543	-2.688 to 2.719	>0.9999
**0 vs IV**	1.016	-0.999 to 3.031	0.4532
**0 vs V**	0.4675	-0.750 to 1.686	0.6165
**0 vs VI**	3.055	1.112 to 4.998	0.0003

**Visuospatial composite**	**Mean difference**	**95% CI of difference**	**Adjusted *p* value**
**0 vs I**	-0.1264	-1.158 to 0.904	0.9983
**0 vs II**	0.3601	-0.419 to 1.140	0.6461
**0 vs III**	0.7250	-2.570 to 4.020	0.8630
**0 vs IV**	0.2478	-1.193 to 1.689	0.9899
**0 vs V**	0.8388	-0.887 to 2.566	0.4229
**0 vs VI**	3.413	1.405 to 5.421	0.0001

MMSE	Mean difference	95% CI of difference	Adjusted *p* value
**0 vs I**	0.1644	-0.657 to 0.986	0.9890
**0 vs II**	0.1561	-0.585 to 0.897	0.9890
**0 vs III**	0.5561	-1.366 to 2.478	0.8628
**0 vs IV**	1.723	0.195 to 3.250	0.0090
**0 vs V**	3.623	1.622 to 5.624	<0.0001
**0 vs VI**	11.02	8.612 to 13.43	<0.0001

MOCA	Mean difference	95% CI of difference	Adjusted *p* value
**0 vs I**	0.1877	-2.883 to 3.259	>0.9999
**0 vs II**	0.8911	-0.522 to 2.305	0.3089
**0 vs III**	4.073	-2.363 to 10.51	0.1540
**0 vs IV**	5.249	1.438 to 9.060	0.0025
**0 vs V**	6.121	1.812 to 10.43	0.0032
**0 vs VI**	14.64	9.706 to 19.58	<0.0001

All analyses conducted with ANOVA (Brown-Forsythe) test. All ANOVAs significant at $p < 0.001$. Adjusted p values reflect p values after multiple comparisons correction with Dunnett's T3 multiple comparisons test.

Years to onset of symptoms: ANOVA: $F = 31.59$; $p < 0.0001$, $R^2 = 0.9186$.

Supplementary Table 7. Clinical, demographic and summary biomarker data for sub-sample with longitudinal tau-PET.

	CU	MCI	*p* value	Alzheimer syndrome	*p* value
**No.**	87	45	—	27	—
**Age, y, mean (SD)**	70.83 (7.16)	70.11 (8.3)	0.61	67.21 (8.7)	0.03
**Female, no. (%)**	53 (61)	24 (53)	0.40	13 (48)	0.40
**Education, y, mean (SD)**	15.20 (3.82)	14.59 (3.61)	0.41	14.28 (4.03)	0.65
***APOE ε4* carriers, %**	24 (28)	16 (36)	0.34	13 (48)	0.04
**MMSE, mean (SD)**	29.06 (1.19)	28.16 (1.59)	<0.0001	21.07 (5.66)	<0.0001
**Neocortical [^{18}F]AZD4694 SUVR (SD)**	1.41 (0.32)	1.91 (0.59)	<0.0001	2.38 (0.59)	<0.0001
**Temporal Meta-ROI [^{18}F]MK6240 (SD)**	1.05 (0.14)	1.39 (0.43)	<0.0001	2.78 (0.96)	<0.0001
**Hippocampal volume, mm^3 (SD)**	3.59 (0.43)	3.21 (0.49)	<0.0001	2.88 (0.54)	<0.0001

p values reported are for comparisons to cognitively unimpaired (CU) subjects. *p* values indicate values assessed with independent samples t-tests for each variable except sex and APOE *ε4* status, where contingency χ^2 tests were performed. MMSE = Mini-Mental State Examination; SUVR = Standardized Uptake Value Ratio; CU = Cognitively Unimpaired; MCI = Mild Cognitive Impairment.
