# Supplementary material for: Biomarker modeling of Alzheimer’s disease using PET-based Braak staging
Source: Nat Aging. 2022 Apr 25;2(6):526–35. doi: 10.1038/s43587-022-00204-0 (PMC10154209; doi:10.1038/s43587-022-00204-0)
Supplement: Supplementary file 2 — Reporting Summary [file 43587_2022_204_MOESM2_ESM.pdf]

Corresponding author(s): Joseph Therriault, Pedro Rosa-Neto

Last updated by author(s): Feb 23, 2022

Reporting Summary

Nature Portfolio wishes to improve the reproducibility of the work that we publish. This form provides structure for consistency and transparency in reporting. For further information on Nature Portfolio policies, see our Editorial Policies and the Editorial Policy Checklist.

Statistics

For all statistical analyses, confirm that the following items are present in the figure legend, table legend, main text, or Methods section.

n/a	Confirmed	
☐	☒	The exact sample size (n) for each experimental group/condition, given as a discrete number and unit of measurement
☐	☒	A statement on whether measurements were taken from distinct samples or whether the same sample was measured repeatedly
☐	☒	The statistical test(s) used AND whether they are one- or two-sided<br>*Only common tests should be described solely by name; describe more complex techniques in the Methods section.*
☐	☒	A description of all covariates tested
☐	☒	A description of any assumptions or corrections, such as tests of normality and adjustment for multiple comparisons
☐	☒	A full description of the statistical parameters including central tendency (e.g. means) or other basic estimates (e.g. regression coefficient) AND variation (e.g. standard deviation) or associated estimates of uncertainty (e.g. confidence intervals)
☐	☒	For null hypothesis testing, the test statistic (e.g. F, t, r) with confidence intervals, effect sizes, degrees of freedom and P value noted<br>*Give P values as exact values whenever suitable.*
☒	☐	For Bayesian analysis, information on the choice of priors and Markov chain Monte Carlo settings
☒	☐	For hierarchical and complex designs, identification of the appropriate level for tests and full reporting of outcomes
☒	☐	Estimates of effect sizes (e.g. Cohen's d, Pearson's r), indicating how they were calculated

Our web collection on statistics for biologists contains articles on many of the points above.

Software and code

Policy information about availability of computer code

Data collection	No software was employed for data collection.
Data analysis	Data was analyzed using R version 3.5.3<br>MRI hippocampal volume data were processed using FreeSurfer.<br>PET images were processed and analyzed in MINC format.

For manuscripts utilizing custom algorithms or software that are central to the research but not yet described in published literature, software must be made available to editors and reviewers. We strongly encourage code deposition in a community repository (e.g. GitHub). See the Nature Portfolio guidelines for submitting code & software for further information.

Data

Policy information about availability of data

All manuscripts must include a data availability statement. This statement should provide the following information, where applicable:

- Accession codes, unique identifiers, or web links for publicly available datasets
- A description of any restrictions on data availability
- For clinical datasets or third party data, please ensure that the statement adheres to our policy

Data availability
All requests for raw and analyzed data and materials will be promptly reviewed by McGill University to verify if the request is subject to any intellectual property or confidentiality obligations. Anonymized data will be shared upon request to the study's senior author from a qualified academic investigator for sole the purpose of replicating the procedures and results presented in this article. Any data and materials that can be shared will be released via a material transfer agreement. Data

are not publicly available due to information that could compromise the privacy of research participants. Related documents including study protocol and informed consent forms can similarly be made available upon request.

Field-specific reporting

Please select the one below that is the best fit for your research. If you are not sure, read the appropriate sections before making your selection.

☒ Life sciences ☐ Behavioural & social sciences ☐ Ecological, evolutionary & environmental sciences

For a reference copy of the document with all sections, see nature.com/documents/nr-reporting-summary-flat.pdf

Life sciences study design

All studies must disclose on these points even when the disclosure is negative.

Sample size	Based on preliminary data supporting a highly conservative Cohen's d of 1.2 for CSF differences, an allocation ratio of 4:1, a power of 95% and an alpha of 0.05, we would need to allocate 10 individuals at Braak stage II to detect CSF differences between Braak 0 and Braak II groups. Based on meta-analyses supporting a Cohen's d of 1.5 for CSF differences (Sunderland et al 2003) between control and Alzheimer's disease groups, an allocation ratio of 4:1, a power of 95% and an alpha of 0.05, we would need to allocate 6 individuals per group (stage IV, stage V and stage VI) to detect CSF differences between Braak 0 and late Braak stage groups.<br><br>Sample sizes were determined based on the availability of amyloid-PET, tau-PET, MRI, CSF biomarkers, and plasma biomarkers. Based on studies of independent populations reporting 12% of late middle age adults without cognitive impairment had detectable tau pathology in Braak II regions (Betthauser et al, Brain 2020), we estimated approximately 25 individuals in the (slightly older) CU elderly population in this study would have detectable tau pathology in Braak II regions. We estimated that over 80% of the AD dementia group (approximately 55 individuals in our study) would demonstrate advanced tau pathology (Braak IV or greater) based on previous studies demonstrating high rates of tau positivity in this group from independent populations.
Data exclusions	We excluded 21 individuals with clinical diagnoses of non-AD neurodegenerative diseases (12 Frontotemporal dementia; 4 Vascular dementia; 2 propressive supranuclear palsy; 1 Corticobasal Degeneration; 1 Hippocampal Sclerosis; 1 Cerebral Amyloid Angiopathy). We also excluded 30 cognitively unimpaired young adults. Data from the young adults was used to determine thresholds for tau-PET abnormality in Braak stages, but they were not included in Alzheimer's disease biomarker modelling analyses. Finally, we did not recruit individuals with inadequately treated systemic medical conditions, active substance abuse, recent head trauma, or MRI/PET safety contraindications. These data exclusion criteria were pre-established.
Replication	A single cohort was used to generate the results obtained in this study; no replication was performed.
Randomization	The data in the present study were collected from a longitudinal observational cohort. There was no allocation to experimental conditions vs other conditions in the present study. Therefore, randomization is not applicable to this study.
Blinding	The Braak staging procedure, where each individual is assigned an individual stage, was carried out blinded to clinical diagnosis and blinded to amyloid-beta status. Clinical diagnoses were carried out before PET imaging. Blinding is not relevant to this study; clinical information was used as an outcome measure and thus investigators could not be blinded to it during analysis.

Reporting for specific materials, systems and methods

We require information from authors about some types of materials, experimental systems and methods used in many studies. Here, indicate whether each material, system or method listed is relevant to your study. If you are not sure if a list item applies to your research, read the appropriate section before selecting a response.

Materials & experimental systems

n/a	Involved in the study	
☒	☐	Antibodies
☒	☐	Eukaryotic cell lines
☒	☐	Palaeontology and archaeology
☒	☐	Animals and other organisms
☐	☒	Human research participants
☒	☐	Clinical data
☒	☐	Dual use research of concern

Methods

n/a	Involved in the study	
☒	☐	ChIP-seq
☒	☐	Flow cytometry
☒	☐	MRI-based neuroimaging

Human research participants

Policy information about studies involving human research participants

Population characteristics	Demographics of all subjects are summarized in Table 1. In brief, the average age of cognitively unimpaired elderly individuals was 71.12 years (sd = 7.18). Average age of individuals with MCI was 70.34 (sd = 8.10) and AD was 66.71 (9.80).

Recruitment	Participants in the TRIAD cohort are enrolled either from the community or outpatients from a memory clinic. Therefore the study made up of individuals who are willing to participate on a study on aging and AD, and correspondingly may not represent the general population.
Ethics oversight	This study was approved by the Montreal Neurological Institute PET working committee and the Douglas Mental Health University Institute Research Ethics Board, and written informed consent was obtained from all research participants.

Note that full information on the approval of the study protocol must also be provided in the manuscript.
